# Acute Aerobic Exercise Based Cognitive and Motor Priming: Practical Applications and Mechanisms

**DOI:** 10.3389/fpsyg.2019.02790

**Published:** 2019-12-12

**Authors:** Terence A. Moriarty, Christine Mermier, Len Kravitz, Ann Gibson, Nicholas Beltz, Micah Zuhl

**Affiliations:** ^1^Department of Health, Exercise, and Sports Sciences, The University of New Mexico, Albuquerque, NM, United States; ^2^Department of Kinesiology, University of Northern Iowa, Cedar Falls, IA, United States; ^3^Department of Kinesiology, University of Wisconsin-Eau Claire, Eau Claire, WI, United States; ^4^School of Health Sciences, Central Michigan University, Mount Pleasant, MI, United States

**Keywords:** priming, exercise, cognition, motor, rehabilitation

## Abstract

Acute exercise stimulates brain regions involved in motor and cognitive processes. Recent research efforts have explored the benefits of aerobic exercise on brain health and cognitive functioning with positive results reported for both healthy and neurocognitively impaired individuals. Specifically, exercise positioned near therapeutic (both behavioral and physical) activities may enhance outcomes associated with treatment outcomes (e.g., depression or motor skill) through neural plasticity promoting mechanisms (e.g., increased brain flow and oxygenation). This approach has been termed “exercise priming” and is a relatively new topic of exploration in the fields of exercise science and motor control. The authors report on physiological mechanisms that are related to the priming effect. In addition, parameters related to the exercise bout (e.g., intensity, duration) and the idea of combining exercise and therapeutic rehabilitation are explored. This exercise-based priming concept has the potential to be applied to many areas such as education, cognitive therapy, and motor rehabilitation.

## Introduction

A lack of cardiovascular fitness has been linked with cognitive dysfunction and learning deficits in various clinical populations (Katz et al., 2012; Alosco et al., 2014); for this reason, recent research efforts have explored the benefits of aerobic exercise on brain health and cognitive functioning with positive results reported for both healthy and neurocognitively impaired individuals (Stoykov et al., 2017). For example, aerobic exercise has been shown to improve memory, processing speed and executive functioning among those with mental deficiencies (Altmann et al., 2016; Zhu et al., 2018), along with facilitating learning in healthy adults (Young et al., 2015; Venckunas et al., 2016; Stern et al., 2019) and adolescents (Berse et al., 2015).

While it appears that aerobic exercise enhances cognitive and motor abilities in humans, it is less well known if gains associated with exercise transfer into improved learning of skills (both motor and cognitive) and learning that many rehabilitation therapies rely heavily upon (e.g., stroke neurorehabilitation, cognitive behavioral therapy). Improved skill learning, such as coping in cognitive behavioral therapy or muscle coordination in physical therapy would ultimately lead to better clinical outcomes. Aerobic exercise may facilitate improvements in treatment outcomes (e.g., abstinence, anxiety, depression, motor skill development) through neural stimulation and plasticity promoting mechanisms (e.g., increased blood flow and oxygenation). Cognitive performance components including information processing and memory may be of particular importance for skill acquisition in various rehabilitation programs (Taubert et al., 2015). Therefore, brain activation through aerobic exercise may lead to downstream retention (i.e., memory recall) of cognitive and motor skills taught during therapy or sport coaching. This would be beneficial for those with mood disorders, substance abuse disorders, stroke patients, athletes, and those with neuromuscular injuries. This concept has been defined as “exercise priming” and involves acute exercise stimulation prior to, or after one’s engagement in therapy or motor skill training (Charalambous et al., 2018). In practice, performing a brief bout of aerobic exercise prior to cognitive or physical therapy, or before a practice session may lead to improvements in therapeutic or practical outcomes.

Efforts to understand the neuropsychological mechanisms of exercise priming is of vital importance to establish exercise as an adjunct treatment for various therapeutic or learning outcomes. Here, we focus on acute aerobic exercise and its priming effects on cognitive function, learning, and motor skill acquisition. For review papers on the benefits of aerobic, resistance, and combined aerobic and resistance exercise on cognitive performance, please see the reviews of Brunt et al. (2019), Landrigan et al. (2019), Smith et al. (2010), Wilke et al. (2019), and Zheng et al. (2016).

## Neuropsychological Mechanisms of Aerobic Exercise Priming

### Brain Blood Flow and Oxygenation

Global brain blood flow remains relatively constant during acute aerobic exercise; although, there may be a shift in resources (i.e., oxygen consumption) from areas required for cognitive function to areas required for motor control and maintenance of vital function (e.g., blood pressure and thermoregulation) (Ide and Secher, 2000; Dietrich and Sparling, 2004). In specific cortical regions, blood flow and oxygenation (i.e., activation) are influenced by the intensity of the exercise bout. For example, activation in the prefrontal cortex (PFC), measured by brain oxygenation, increased during submaximal aerobic exercise (up to 80% of peak ability) but then decreased when intensity reached very hard or maximal effort (Rooks et al., 2010).

Upon cessation of low-moderate intensity aerobic exercise, cerebral oxygenation remains elevated for up to 30 min (Faulkner et al., 2016; Stavres et al., 2017; Tsubaki et al., 2018). Performing a cognitive task after exercise is potentially ideal as it takes advantage of the heightened cortical activity during recovery. Twenty to 30 min of moderate intensity (50–60% VO_2__max_) cycling increased post-exercise cortical oxygenation (i.e., activation), which aligned with improvements in post-exercise executive function performance (Stroop task) (Yanagisawa et al., 2010; Stavres et al., 2017; Tsubaki et al., 2018). Increased PFC oxygenation may be indicative of higher cortical activity and, therefore, greater mental effort leading to improved cognitive processes such as working memory and attention (Herold et al., 2018). However, a negative association between left-PFC activation and processing speed has been recently reported among middle aged adults after acute bouts of both low and high intensity aerobic exercise, along with yoga (Moriarty et al., 2019). This acute response may be interpreted as increased neural efficiency, more specifically defined as reduced mental input (lower PFC activation) for mental processing (Causse et al., 2017). The differing results might be in response to the cortical region monitored and the cognitive task performed. For example, neural efficiency improvements for processing speed indicates less PFC neural input required to process information (Rypma et al., 2006). Higher PFC activation during executive function tasks is logical because these measures require memory and attention (Albinet et al., 2014).

In summary, evidence suggests that cerebral blood flow rises during low- to moderate-intensity exercise, and translates to post-exercise alterations in PFC activity, which lead to improvements in various cognitive domains (e.g., executive function and processing speed).

### Plasticity and Neurotrophic Factors

Acute exercise can also enhance the immediate induction of markers of brain plasticity. More specifically, a single 20–30-min bout of moderate and high intensity aerobic exercise led to a transient decrease in short-interval intracortical inhibition and M1 excitability, which are indicators of plasticity in the motor cortex (Smith et al., 2014; Mang et al., 2016a; Singh et al., 2016; Neva et al., 2017). Exercise-induced plasticity has been linked to improvements in cognitive function, such as processing speed (Singh et al., 2016). The plasticity mechanism may be through exercise-induced release of neurotrophins (McDonnell et al., 2013). For the purpose of this review we have decided to focus on two neurotrophins, vascular endothelial growth factor (VEGF) and brain-derived neurotrophic factor (BDNF), both of which have been shown to be upregulated after various types of physical exercise (Maass et al., 2016; Heisz et al., 2017).

Vascular endothelial growth factor is a well-known growth factor and an important signaling molecule involved in angiogenesis and vasculogenesis (Amaral et al., 2001; Lee and Son, 2009). Interestingly, VEGF-A, which is a gene from the VEGF family, increased following either high-intensity exercise or a lactate injection among C57BL/6 mice (Lezi et al., 2013). Gustafsson et al. (1999) also reported that a single bout of dynamic exercise increased VEGF and that there was a graded VEGF response directly related to the metabolic stress of exercise in humans. Therefore, it has been proposed that the VEGF response is from lactate production during exercise. It has been suggested that activation of the lactate receptor (HCAR1) in the brain enhances the effect of VEGF-A and brain angiogenesis, thereby providing a link between aerobic exercise and brain nourishment (Morland et al., 2017). Lactate has also been linked with promoting the expression of plasticity genes (including BDNF) and being required for long-term memory formation and processing (Newman et al., 2011; Schiffer et al., 2011; Suzuki et al., 2011; Yang et al., 2014). Moreover, intravenous infusion of 100 mM L-lactate has been shown to ameliorate cognitive impairment in rats after traumatic brain injury (Holloway et al., 2007). Since brain dysfunctions are associated with hypoperfusion and vascular complications, lactate release as a result of exercise may facilitate VEGF expression and act as a potential mechanism for treatment against cognitive decline and other brain conditions.

In addition, BDNF is emerging as a key mediator of synaptic plasticity in the central computational hub for memory processing (i.e., the hippocampus), and is thought to be modulated by insulin growth factor-1 (IGF-1) (Ding et al., 2006). At the cellular level, increases in BDNF may be the link between exercise and learning; however, BDNF induction in response to acute exercise is mixed, and may be influenced by the intensity of exercise, along with the level of cognitive impairment among research participants (Charalambous et al., 2018). BDNF is thought to regulate synaptic proteins (e.g., synapsin I and synaptophysin) within the hippocampus thereby improving axonal branching and allowing for an increased effectiveness in synaptic transmission (Danzer et al., 2002; Vaynman et al., 2004). Korte et al. (1995) blocked the expression of BDNF in mice and found them to have a significantly reduced long-term potentiation (a measure of synaptic plasticity). Heldt et al. (2007) found that BDNF deletion from the hippocampus impaired novel object recognition and spatial learning in mice. Importantly, these impairments are reversed when exogenous BDNF is given to a BDNF-deficient animal (Patterson et al., 1996), further providing support for the importance of this neurotrophic factor in neural and cognitive function (Cotman and Berchtold, 2002).

In humans, serum BDNF is commonly measured as an indirect indicator of neurogenesis. This is based on evidence that BDNF produced in the brain accounts for 70–80% of circulating BDNF in response to aerobic exercise in humans (Rasmussen et al., 2009). The increase in serum BDNF has been reported in response to acute bouts of aerobic exercise and also linked with better hippocampal function (Griffin et al., 2011). The magnitude of the increase in serum BDNF in humans may be exercise intensity dependent. Ferris et al. (2007) reported a 13 and 30% increase in serum BDNF following cycling above ventilatory threshold or graded exercise test to volitional fatigue, respectively. More recently, Ross et al. (2019) reported a direct linear relationship between exercise intensity and post-exercise serum BDNF among both healthy and moderately depressed individuals. The intensity-dependent increase in serum BDNF has also been positively associated with improved prefrontal cognitive functioning in humans (Hwang et al., 2016). Conversely, Tsai et al. (2014, 2016, 2018) found no relationship between increased BDNF concentrations and improved cognitive performance following 30-min of moderate-intensity exercise in both healthy adults and older adults with mild cognitive impairment. Accordingly, these results purport that the circulating BDNF response to exercise is not linked to post-exercise cognitive performance. The transient increase in BDNF after acute exercise may explain the lack of correlations with cognitive performance. Therefore, it is possible that the post-exercise timing of the serum BDNF measurements align with the cognitive testing influenced outcomes (Tsai et al., 2018). Alternately, non-BDNF mechanisms (neurotrophins, arousal, hormones) could be responsible for the cognitive changes after exercise.

While conflicting, these data indicate that acute aerobic exercise can improve cognitive function concomitant with increased serum BDNF concentrations, thereby suggesting a functional role for this neurotrophic factor in acute exercise-induced cognitive enhancement in humans. However, the BDNF changes may not be the only potential factor that drives improvements in cognitive performance after aerobic exercise.

In summary, motor plasticity in response to acute aerobic exercise may induce the release of neurotrophins, VEGF and BDNF, which, are linked to cognitive performance improvements.

### Neuroendocrine and Myokines

Catecholamines, such as norepinephrine (NE) and dopamine (DA) have been attributed to the cognitive benefits of acute exercise, which has been defined as the “catecholamine hypothesis” (McMorris, 2016). According to this model, acute short duration, moderate intensity exercise stimulates NE synthesis in the PFC region leading to increased arousal and attention. Conversely, during long duration, high intensity exercise, a larger release of NE, along with DA dampen neuronal activity causing a decline in executive functioning (McMorris, 2016). However, NE released during long duration, moderate intensity aerobic exercise has been shown to facilitate the synthesis of brain BDNF (McMorris, 2016). Thus, catecholamine release supports brain function after moderate intensity exercise, regardless of duration.

Acute aerobic exercise at approximately 60% intensity, or higher stimulates the hypothalamic-pituitary-adrenal (HPA) axis and increases the secretion of cortisol, which peaks around 30 min post exercise and remains elevated for 2 h (Duclos et al., 1998; Hill et al., 2008). Evidence suggests that the cortisol released improves learning and memory by interacting with glucocorticoid and mineralocorticoid receptors located in the hippocampus, amygdala, and PFC regions of the brain (Heffelfinger and Newcomer, 2001; Yuen et al., 2009). A positive correlation was detected between the increase in cortisol release after exercise and vocabulary retention among healthy adults (Hötting et al., 2016). Interestingly, cortisol released in response to a psychosocial stress task resulted in an impaired retrieval of words (Tollenaar et al., 2008). The differing memory responses may be due to the higher cortisol released during the psychological stress compared to physical stress. Large increases in cortisol impairs memory, thus indicating a possible threshold for cortisol mediation of cognitive function (Dominique et al., 2000; Basso and Suzuki, 2017).

Myokines, which are released from skeletal muscle, may also help to explain the cognitive and motor functioning benefits of acute exercise (Kim et al., 2019). Of note, irisin has been shown to attenuate brain damage incurred during various types of cerebral insult (ischemia, stroke) (Asadi et al., 2018). Irisin functions via activation of Akt and ERK1/2 pro-survival signaling pathways thereby reducing ischemia-induced neuronal injury (Li et al., 2017). In addition, aerobic exercise increased the expression of FNDC5 (a membrane protein that is cleaved and secreted as irisin), which led to upregulation of BDNF in the hippocampus, thus demonstrating a link between exercise-induced irisin and neurogenesis (Wrann et al., 2013). Cathepsin B is another recently identified myokine that is important for neural plasticity and cognitive function (Moon et al., 2016). Aerobic exercise has been shown to increase cathepsin B plasma levels in mice, monkeys, and humans (Moon et al., 2016). In humans, changes in cathepsin B positively correlated with fitness and memory (Moon et al., 2016). Though, to date, little research has been conducted on myokines’ effect on the brain; however, both irisin and cathepsin B may play important roles in the beneficial effects of exercise on brain health and function.

In summary, acute aerobic exercise appears to promote cognitive gains which may, in part, be mediated through cerebral blood flow and cortical activation, growth and neurotrophic factors, as well as hormones and myokines. For this reason, researchers are beginning to examine the role of exercise as a cognition-stimulating mechanism to improve cognitive performance and enhance motor skill acquisition in both healthy and clinical populations (Lefferts et al., 2019).

## Acute Aerobic Exercise as a Priming Technique for Cognitive Improvements and Motor Skill Acquisition

“Exercise priming” refers to a non-conscious process that promotes cognitive or motor skill related learning, whereby performing an acute exercise bout alters the response of another stimulus (Stoykov et al., 2017). Priming relies on the transient cognitive benefits of acute aerobic exercise and, when strategically performed before or after a task (either motor or cognitive), may improve learning outcomes. In humans, commonly studied cognitive domains include information processing, reaction time, memory, executive functioning, and attention (Chang et al., 2012). Typical study protocols include a baseline measurement of the specific cognitive domain(s) or motor task of interest followed by an acute aerobic exercise session and then retesting of the cognitive domain(s) or motor task. Acute bouts of aerobic exercise ranging from 10 to 30 min at an intensity of 40–100% of maximal intensity have stimulated improvements in various cognitive domains and motor tasks.

For example, young adults demonstrated improved reaction times after performing acute moderate-intensity (50–70% of maximal heart rate) exercise for 20–30 min (Sibley et al., 2006; Harveson et al., 2016; Wang et al., 2019). Cognitive improvements have also been reported among clinical populations (e.g., patients with Multiple Sclerosis, depression, breast cancer) after acute bouts of moderate-intensity activity (Vasques et al., 2011; Sandroff et al., 2015; Salerno et al., 2019). Recently, Lefferts et al. (2019) found that a 30-min bout of moderate-intensity cycling decreased reaction time in the Eriksen Flanker task and in a memory recognition task in middle-aged individuals with hypertension. Support for the “priming” effect on behavioral performance has also been illustrated in post-stroke patients following an acute 15-min bout of cycling as reported by an improvement in the behavioral performance of a working memory task compared with the non-exercise control condition (Moriya et al., 2016). In addition, acute short duration (15–20 min) exercise has also been shown to improve motor skill learning and performance (Roig et al., 2012; Mang et al., 2014, 2016b; Skriver et al., 2014; Stavrinos and Coxon, 2017; Dal Maso et al., 2018). For example, Mang et al. (2016b) and Roig et al. (2012) found that 20 min of high-intensity interval cycling (3-min at 90% VO_2__peak_ alternating with 3-min recovery) performed prior to motor skill practice, improved memory recall of the skill in response to a 24-h delayed retention test. In addition, several research groups have reported improved motor skill retention when exercise is performed after practicing the motor skill (Roig et al., 2012; Stavrinos and Coxon, 2017; Dal Maso et al., 2018). The authors suggested the improvement occurred as a result of an increased rate of motor memory retrieval and learning (Mang et al., 2014, 2016b). More recent evidence has also found that the effects of performing exercise after practicing a motor task are beneficial for retention of that particular task (Stavrinos and Coxon, 2017; Dal Maso et al., 2018). Conversely, no improvements in cognitive and motor function after acute exercise has also been reported (Thomas et al., 2017; Moriarty et al., 2019). The null findings have been attributed to the exercise stimulus administered and also to the timing of cognitive or motor testing (Chang et al., 2012; Thomas et al., 2016). A greater stimulus (more intense exercise), along with administering testing at least 20 min post-exercise appear to influence cognitive and motor results (Chang et al., 2012). Other reported factors, include cognitive or motor construct measured, and the health status of the participants (Chang et al., 2012). In addition, previous research in both adolescent and adult populations have argued that those with higher fitness levels demonstrate greater neural efficiency, and in turn, greater improvements in cognitive performance (Stroth et al., 2009; Renaud et al., 2010; Chan et al., 2011; Hogan et al., 2013). In summary, a fair amount of evidence suggests that acute exercise leads to cognitive improvements and motor skill acquisition among young and older adults, along with clinical populations.

**FIGURE 1:**
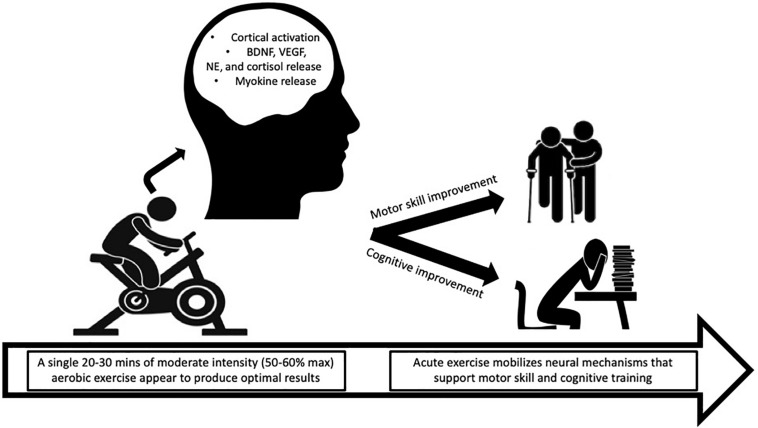
An acute 20–30-min bout of moderate-intensity aerobic exercise may mobilize neural mechanisms that help prime the brain for cognitive and motor performance. BDNF, brain-derived neurotrophic factor; VEGF, vascular endothelial growth factor; NE, norepinephrine.

The evidence presented above suggests that acute exercise positioned proximal to (before or after) cognitive or motor tasks leads to performance improvements (see Figure 1). Based on these data, exercise may serve as an adjunctive therapy to cognitive or motor (i.e., physical) therapies. Examples include an aerobic exercise program designed to operate alongside cognitive behavioral therapy (CBT) for treatment of substance use disorder or severe depression or an aerobic exercise program in conjunction with physical therapy for stroke patients. Early onset of cognitive deficits can signal brain and behavioral disorders such as schizophrenia and Alzheimer’s disease (Uhlhaas and Singer, 2006). The cognitive or motor function improvements induced by exercise may “prime” the patient to more fully engage in and benefit from therapeutic tasks for various treatments. Limited efforts have been made to partner exercise with therapy; whereas, the bulk of research has compared exercise alone to therapy (Stathopoulou et al., 2006; Zschucke et al., 2013). We argue that exercise cannot replace cognitive or motor therapy but may be able to enhance the benefits. For example, exercise combined with cognitive therapy for the treatment of schizophrenia improved symptoms and functional outcomes compared to cognitive therapy alone (Malchow et al., 2016; Nuechterlein et al., 2016). In addition, stimulant users report more abstinent days when they participate in exercise combined with therapy compared to therapy partnered with health education (Trivedi et al., 2017).

In summary, both acute and chronic exercise training studies have provided initial evidence that aerobic exercise improves cognitive and motor function in cognitively impaired individuals. Specifically, the cognitive benefits associated with such exercise interventions have been shown to extend beyond improving mobility of limbs to also improving sensorimotor learning and performance in cognitive tasks. Therefore, aerobic exercise in combination with therapeutic recovery and motor control rehabilitation techniques may ultimately augment functional outcomes through cognitive effects. As the number of individuals with cognitive and motor impairments continue to rise, it is imperative that more research is conducted in this area to define the specific parameters (e.g., intensity, duration) and combinations (e.g., exercise and therapy) of such chronic exercise interventions.

## Conclusion

Aerobic exercise, both acute and chronic, has the ability to prime the brain for both cognitive and motor task performance. These findings provide a stable groundwork for designing and prescribing acute aerobic exercise in future research studies examining the effects of exercise on cognitive performance and motor skill acquisition. Applying this priming idea to education, rehabilitation, and therapy has the potential for improved cognitive and motor performance and may form an important component of improvements in these fields. Finally, the combination of exercise and various forms of therapeutic rehabilitation may enhance the functional outcomes and quality of life for individuals with cognitive or motor impairments and perhaps be the way of the future with further in-depth research and knowledge of mechanisms.

## Author Contributions

TM contributed to writing the first draft of the manuscript, conception and design of the manuscript, and revising the manuscript critically. CM, LK, AG, and NB contributed to revising the manuscript critically for important intellectual content, initiating the idea, and monitoring progress. MZ contributed to writing the manuscript, conception and design of the manuscript, and monitoring progress. All authors contributed to the manuscript revision, and read and approved the submitted version.

## Conflict of Interest

The authors declare that the research was conducted in the absence of any commercial or financial relationships that could be construed as a potential conflict of interest.
